# Exploring the Incorporation of a Positive Psychology Component in a Cognitive Behavioral Internet-Based Program for Depressive Symptoms. Results Throughout the Intervention Process

**DOI:** 10.3389/fpsyg.2018.02360

**Published:** 2018-11-29

**Authors:** Adriana Mira, Juana Bretón-López, Ángel Enrique, Diana Castilla, Azucena García-Palacios, Rosa Baños, Cristina Botella

**Affiliations:** ^1^Department of Psychology and Sociology, Universidad de Zaragoza, Teruel, Spain; ^2^Department of Basic Psychology, Clinic and Psychobiology, Universitat Jaume I, Castellón, Spain; ^3^CIBER Fisiopatología Obesidad y Nutrición (CIBERobn), Instituto Salud Carlos III, Santiago de Compostela, Spain; ^4^E-mental Health Research Group, School of Psychology, University of Dublin, Trinity College, Dublin, Ireland; ^5^Department of Personality, Evaluation and Psychological Treatment, Universidad de Valencia, Valencia, Spain

**Keywords:** depressive symptoms, positive psychology, internet-based Intervention, intervention process, post-module assessment

## Abstract

Traditionally, evidence-based treatments for depression have focused on negative symptoms. Different authors describe the need to include positive affect as a major target of treatment. Positive psychology aims to fill this gap. Reaching everyone in need is also important, and Internet-based interventions can help in this task. The present study is a secondary analysis derived from a randomized controlled trial aimed to test the efficacy of an Internet-based intervention for patients with depressive symptoms. This intervention consisted of an 8-module Internet-based program that combined four modules based on cognitive-behavioral therapy strategies and four modules based on positive psychology strategies. The main goal of this secondary analysis is to report the data collected after each module from the participants who completed the intervention, explore the changes throughout the intervention process, and examine the changes observed in the different variables before versus after the introduction of the positive psychology component. A total of 103 patients completed the intervention. At pre-, post-intervention, and post-module evaluations, they completed positive and negative affect, depression, and anxiety measures. Negative affect and anxiety decreased significantly during the implementation of the cognitive-behavioral therapy and positive psychology modules. However, depression and positive affect improved only after the introduction of the positive psychology modules. This is the first study to explore, throughout the intervention process (module by module), the incorporation of a positive psychology component in an Internet-based program. Results suggest that positive psychology techniques might have an impact on clinical symptomatology, and they emphasize the need to include these techniques to achieve a more profound change in positive functioning measures.

**Clinical Trial Registration:** NCT02148354 (http://ClinicalTrials.gov/ct2/show/NCT02148354).

## Introduction

Depression is one of the most common health problems worldwide. Its economic cost is quite high, and it disrupts the lives of millions of people each year (Haro et al., 2014). The increasing prevalence of this disease and its tendency toward chronicity suggest that its prevention and treatment should be a health priority (Ferrari et al., 2013; Haro et al., 2014).

Evidence-based psychological interventions for depression have been shown to be effective (Nathan and Gorman, 2015). However, these interventions are usually designed to reduce negative symptoms and deficits rather than building positive resources (Dunn, 2012). In this regard, the absence of mental illness does not necessarily imply the presence of well-being (Gotlib and Hammen, 2008; Keyes and Simoes, 2012). The literature emphasizes the role of both negative and positive affect in the initiation and maintenance of emotional disorders (Kessler et al., 2011; Barlow et al., 2013). Moreover, depressive symptoms often involve low levels of positive emotions, engagement, and purpose in life, but they are typically viewed as consequences or mere correlates of depression (Ruini, 2017). Thus, studies have shown that low levels of positive affect are more strongly linked to depression than to other emotional disorders (Watson and Naragon-Gainey, 2010). Therefore, different authors state that there is a need to include positive affect as a main target of treatment (Werner-Seidler et al., 2013).

Positive psychology (PP) aims to fill this gap by exploring the conditions and processes that contribute to the flourishing or optimal functioning of people, groups, and institutions (Gable and Haidt, 2005). Meta-analyses have shown that positive psychological interventions (PPIs) are effective in enhancing well-being and reducing depressive symptoms (Sin and Lyubomirsky, 2009; Bolier et al., 2013). Despite these findings, some authors claim that there has been an artificial separation between positive and clinical psychology, and they defend the need for an integration of these two approaches in order to diminish suffering and increase well-being, suggesting the term “Positive Clinical Psychology” (Johnson and Wood, 2015). However, few studies have tested the efficacy of treatments combining PPI and Cognitive Behavioral Therapy (CBT) components in clinical settings (Meyer et al., 2009; Bolier et al., 2013; Titov et al., 2013). Furthermore, although the importance of including assessments during the course of treatment is well known (Kazdin, 2007), the majority of studies report pre to post intervention changes, limiting the possibility of analyzing the impact of each specific component.

Another important issue in depression is reaching everyone in need. Internet interventions may be more affordable and accessible than face-to-face interventions (Richards and Richardson, 2012; Kazdin and Rabbitt, 2013; Kazdin, 2015). Furthermore, research results encourage the use of online questionnaires (Carlbring et al., 2007; Vallejo et al., 2007; Hedman et al., 2010) because they offer many advantages over traditional data collection methods. Missing data can be handled better, and scoring is easy and immediate, for example, when patients finish one psychological component (Carlbring et al., 2007), making it possible to discover the specific contribution of each component throughout the intervention process. In addition, online assessment allows users to receive feedback about their progress (Barak and English, 2002).

The combination of Internet and PP allows the emergence of self-help online PP interventions that may contribute to improving individuals’ mental health by offering them a way to self-manage their well-being (Mitchell et al., 2010; Bolier et al., 2013). Therefore, PPIs in a self-help format may be an effective and suitable way to reach a large number of people (Mitchell et al., 2010). These programs can be considered “Positive Technology” interventions, that is, technology-based strategies to improve the quality of the personal experience, increase wellness, and generate strengths and resilience in individuals (Botella et al., 2012; Riva et al., 2012).

To our knowledge, no studies have explored the evolution of both clinical and positive variables throughout an Internet-based positive clinical psychology intervention, including post-module assessments.

The present study is a secondary analysis derived from a randomized controlled trial (RCT) designed to test the efficacy of a positive clinical psychology intervention implemented through positive technology for patients with depressive symptoms (Mira et al., 2017). This intervention consisted of an 8-module Internet-based program that combined 4 modules based on CBT strategies and 4 modules based on PP strategies. In the RCT, overall the results produced medium to large effect sizes compared to the control group, not only in anxiety, depression, and negative affect, but also in positive affect (Mira et al., 2017). However, the contribution of each module to the improvement of these variables is not known; nor is the specific contribution of the PP component because 4 of the 8 modules included PP strategies. Therefore, the main goal of this secondary analysis is to report the data collected after each module from the participants who completed the intervention, explore the changes in positive and negative affect and depression and anxiety symptoms throughout the intervention process, and examine the changes observed in these variables before and after the introduction of the PP component, in order to provide preliminary evidence about the specific contribution of each component. Furthermore, given that some findings point out that depression status moderates the effectiveness of PPIs (Sin and Lyubomirsky, 2009), a second goal of the present study is to explore whether the severity of the depressive symptoms is related to the benefits in positive affect obtained from the PP component.

## Materials and Methods

### Research Design

This is a secondary analysis study derived from an RCT with three independent groups: (a) Internet-based intervention group with automated support (automated mobile phone messages, automated emails, and continued feedback through the program); (b) Internet-based intervention group with automated support plus human support (brief weekly support phone call without clinical content); and (c) Waiting list control group (participants completed the intervention program after the waiting time) (Mira et al., 2017; ClinicalTrials.gov ID: NCT02148354).

All the participants who completed the Internet-based intervention filled out the pre-treatment evaluation through the web platform. After completing each of the eight treatment modules, they filled out the post-module evaluation, also through the web system. At the end of treatment, they also completed the post-treatment evaluation through the web site. Regarding platform usage, participants progressed sequentially through the intervention program in a completely self-applied way over the Internet at their own pace. Participants were encouraged to complete about one module per week in order to obtain maximum benefits from the program. The participants had up to 12 weeks to complete the eight modules.

More details about the design, procedure, therapists, recruitment methods, and support offered to participants are included in the main outcome study (Mira et al., 2017).

### Inclusion Criteria

Inclusion criteria were: age between 18 and 65 years, willingness to participate in the study, ability to use a computer and having an Internet connection at home, ability to understand and read Spanish, currently experiencing at least one stressful event in their lives that produces interference, and having minimal, mild, or moderate depressive symptoms [score of 28 or less on the Beck Depression Inventory-II (BDI-II)].

### Participants

For this study, all the participants from the RCT who completed the Internet-based intervention were included, that is, participants from both intervention groups, with and without human support (no differences were found between them on any measure; Mira et al., 2017), and participants from the waiting list control group who completed the program after the wait time. As a result, 103 participants are included in these analyses.

The sample was composed mostly of women (68%). Regarding marital status, 53.4% of the participants were single, 39.8% married or with a partner, and 6.8% separated or divorced. Regarding the study level, most of the participants had higher education (69.9%), 25.2% had mid-level studies, and the rest had basic studies (4.9%). Ages ranged between 20 and 58 years, with a mean of 35 years (*SD* = 9.42). Regarding depression severity at baseline, the average on the Beck Depression Inventory-II (BDI-II) was 9.50 (*SD* = 7.08).

### Intervention and Protocol Modules

We developed a manualized treatment protocol that included traditional therapeutic components of evidence-based treatments for depression (Motivation, Psychoeducation, Cognitive Therapy, and Behavioral Activation). The program also included a PP component, offering strategies to promote psychological strengths and enhance positive mood. We adapted the protocol to an Internet-based, multimedia interactive program. It is designed for optimal use on a PC, but it can also be used on a tablet.

The intervention protocol consists of eight interactive modules. Four of them are based on CBT, and the other four on PP psychology.

The main objectives of the 4 CBT modules: (1) “*Motivation for change*” (2) “*Understanding problems*” (3) “*Learning to move on*” and (4) “*Learning to be flexible*” are, respectively: (a) to analyze the advantages and disadvantages of obtaining a therapeutic change; (b) to provide information so that the user can understand the nature of emotional problems; (c) to teach the importance of “moving on” in order to acquire a proper level of activity and involvement in life; and (d) to teach a more flexible way of thinking.

Regarding the PP modules, designed to improve well-being and encourage psychological strengths and positive emotionality (see Table 1): (5) “*Learning to enjoy*” (6) “*Learning to live*” (7) “*Living and learning*” and (8) “*From now on, what else…*?”, the main objectives, respectively, are: (a) To promote involvement in pleasant and significant activities, having contact with other people, enjoying positive experiences, and “savoring” positive aspects of life; (b) To understand the importance of identifying the individual’s own psychological strengths and carrying out meaningful activities linked to values and goals in life; (c) To develop an action plan to boost the individual’s psychological strengths and start working for life and the future; and (d) To learn that the end of the program is only the beginning of each person’s path, inviting them to think about how they would like their future life to be, following the positive life mottos (e,g., *Every morning, a new day full of possibilities begins for you; The richest person is the one who knows how to enjoy the best pleasures without spending a penny*).

**Table 1:** Description of the PP modules.

Module	Specific strategies	Empirical source
“Learning to enjoy”	The role of positive emotions in our lives	Ekman et al., 1990; Soussignan, 2002; Miles and Johnston, 2007; Bryant et al., 2011; Fredrickson, 2013
	The importance of the Duchenne smile	
	The concept of ”savoring” and the importance of ”small things”	
“Learning to live”	The importance of identifying the individual’s own psychological strengths	Peterson and Seligman, 2004; Seligman et al., 2005; Ryff, 2014
	The concept and dimensions of well-being	
	Select and record activities linked to values and significant areas and goals in life	
“Living and learning”	Strategies to develop our strengths:
	Gratitude, Curiosity and Hope Identify episodes of wellbeing and maintain them	Fava, 1999; Lee Duckworth et al., 2005; Seligman et al., 2005; Sheldon and Lyubomirsky, 2006
“From now on, what else…?”	What have I learned?	Sheldon and Lyubomirsky, 2006
	How do I want my future to be?: Following positive life mottos and my best possible self exercise	


For more information about the specific content in each module, see Mira et al., 2017.

### Measures

*Severity of depression measure* (at pre-treatment, via the Internet):

The BDI-II is a 21-item self-report scale of depressive symptoms (Beck et al., 1996). It has shown good psychometric properties in several studies (Storch et al., 2004). The Spanish version of the BDI-II was used (Sanz et al., 2005). It shows good reliability and validity data and provides a bifactorial solution that matches what was found in previous studies (Sanz et al., 2005). It has shown high internal consistency in both general (α = 0.87) and clinical populations (α = 0.89) (Sanz et al., 2005).

*Self-assessment measures* (at pre-treatment, post-treatment, and post module evaluation, via the Internet):

Primary outcome measure: Positive and Negative Affect Scale (PANAS) (Watson et al., 1988). It consists of 20 items that evaluate two independent dimensions: positive affect (PA) and negative affect (NA). The range for each scale (10 items on each) is from 10 to 50 (Watson et al., 1988). It is a brief, reliable, and valid self-report measure. It has shown excellent convergent and divergent validity (Watson et al., 1988). As in the original version, the validation of the Spanish PANAS revealed a robust and stable two-dimensional structure, and provided strong support for its validity and reliability (internal consistency: 0.89–0.91 for PA and NA in women and 0.87 for PA and 0.89 for NA in men) (Sandín et al., 1999).

Secondary outcome measures: Overall Anxiety Severity and Impairment Scale (OASIS) (Norman et al., 2006). It consists of 5 items that measure the frequency and severity of anxiety, as well as the level of avoidance, work/school/home interference, and social interference associated with anxiety. It was found to have excellent test–retest reliability, in addition to good convergent and discriminant validity and high internal consistency (α = 0.80) (Norman et al., 2006). The range for the scale is from 0 to 20. The validation data for the Spanish version confirmed the factorial structure and the reliability and validity data obtained by the original authors (Mira et al., 2015).

Overall Depression Severity and Impairment Scale (ODSIS) (Bentley et al., 2014). It is a self-report measure with 5 items that evaluate experiences related to depression. The ODSIS measures the frequency and severity of depression, as well as the level of avoidance, work/school/home interference, and social interference associated with depression. The range for the scale is from 0 to 20. It has shown good convergent and discriminant validity and excellent internal consistency (α = 0.94 in an outpatient sample, 0.91 in a student sample, and 0.92 in a community sample) (Bentley et al., 2014). The validation data for the Spanish version confirmed the factorial structure and the reliability and validity data obtained by the original authors (González-Robles et al., 2015).

### Statistics and Data Analysis

In order to enhance the power of the results, intent-to-treat analyses were carried out to handle missing data. These analyses made it possible to use all the available data collected from the whole sample of participants across the different time points. The procedure was based on the guidelines suggested by Hair et al. (2014). First, the type of missing data was analyzed, concluding that these data were missing at the item level and eligible for imputation. Second, the quantity of missing data was explored, determining that the total amount of missing values was less than 10%. Third, the random pattern of missing data was explored through the Little MCAR test X2 (108) = 108.63, *p* = 0.46 (Little and Rubin, 1990), concluding that missing data were due to chance, and not to any other specific factor. Finally, missing values were imputed through maximum likelihood (ML) estimation procedures. Sensitivity analyses comparing the results of completers and the ITT sample were conducted, determining that both samples followed the same patterns, and concluding that there was no chance of making biased estimations.

In order to explore the change on the OASIS, ODSIS, and PANAS over time, a repeated-measures ANOVA including the 10 temporal assessments (8 modules plus pre- and post-treatment assessment) was conducted. Next, in order to explore the specific contribution of the CBT and PP components, another ANOVA was conducted with three temporal assessments: pre, post-module 4 (after CBT component), and post-treatment (after PP component). Sidak’s *post hoc* analyses were conducted to explore pairwise comparisons. Finally, a univariant MANOVA with the whole sample was conducted with depression severity symptoms as independent variable and the change in positive affect after the presentation of the CBT component and the same change after the presentation of the PP component as outcomes. All statistical analyses were conducted by using IBM SPSS Statistics 20 (IBM Corporation, Armonk, NY, United States).

## Results

### Changes in the Variables Throughout the Intervention Process: Positive and Negative Affect, Depression, and Anxiety

The means and standard deviations presented by the participants on each measure assessed throughout the intervention process are presented below in Table 2. Repeated-measures ANOVA analyses revealed a significant time effect on all measures: PANAS + [*F*_(1,102)_ = 6.63, *p* < 0.001], PANAS – [*F*_(1,102)_ = 19.03, *p* < 0.001]; OASIS [*F*_(1,102)_ = 22.81, *p* < 0.001] and ODSIS [*F*_(1,102)_ = 12.43, *p* < 0.001]. These results indicate that a significant change over time was observed in the scores on the different measures.

**Table 2:** Means and standard deviations in each measure assessed throughout the intervention process.

	CBT therapy modules (M1–M4)										PP therapy modules (M5–M8)									
		
Va.	Pre.		M1		M2		M3		M4		M5		M6		M7		M8		Post	
										
	*M*	*SD*	*M*	*SD*	*M*	*SD*	*M*	*SD*	*M*	*SD*	*M*	*SD*	*M*	*SD*	*M*	*SD*	*M*	*SD*	*M*	*SD*
PA +	28.90	7.65	28.29	8.97	29.09	8.47	28.71	8.54	28.29	9.19	29.32	8.71	29.14	8.07	29.65	8.19	31.57	7.99	31.88	8.14
NA -	18.85	6.38	16.38	5.99	15.54	5.34	15.85	5.89	15.66	5.75	14.53	4.96	14.36	4.61	14.85	5.21	13.68	3.78	13.72	3.75
OA	4.29	3.56	4.01	3.37	2.93	2.99	2.79	2.97	2.86	2.94	2.32	2.74	2.28	2.82	2.36	2.79	1.57	2.21	1.40	1.97
OD	2.81	3.25	2.86	3.30	2.24	2.76	2.36	2.96	2.31	2.98	2.14	3.00	1.81	2.60	1.58	2.29	0.94	1.76	0.92	1.70


*Va., variables; M, mean: SD, standard deviation; Pre, pre-treatment evaluation; Post, post-treatment evaluation; MX, post-module X evaluation (X = 1–8); PA^+^, Positive Affect Scale; NA^-^, Negative Affect Scale; OA, Overall Anxiety Severity and Impairment Scale; OD, Overall Depression Severity and Impairment Scale.*

### Changes in the Variables Before Versus After the Introduction of the PP Modules: Positive and Negative Affect, Depression, and Anxiety

The analysis revealed a significant time effect on all measures: PANAS + [*F*_(1,102)_ = 11.73, *p* < 0.001], PANAS – [*F*_(1,102)_ = 42.08, *p* < 0.001]; OASIS [*F*_(1,102)_ = 26.56, *p* < 0.001] and ODSIS [*F*_(1,102)_ = 19.31, *p* < 0.001].

**FIGURE 1:**
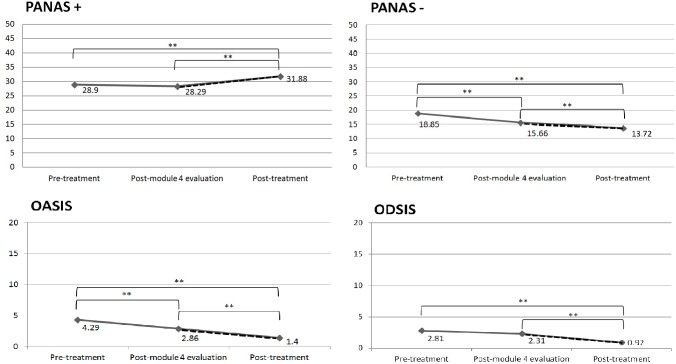
Changes in the variables before versus after the introduction of the PP modules. The dashed line indicates the PP modules; ^∗∗^*p* < 0.01.

The results of the Sidak’s *post hoc* tests are presented in the figures below (see Figure 1). Figure 1 shows that in the case of the PANAS + and the ODSIS, there were significant improvements from the pre- to post-treatment evaluation and from the post-module four (after the CBT component) to post-treatment evaluation (after the PP component). In the case of the PANAS – and the OASIS, there were significant improvements from the pre-treatment evaluation to the post-treatment evaluation, from the pre-treatment evaluation to the post-module four evaluation, and from the post-module four evaluation to the post-treatment evaluation.

### How Depression Severity at Pre-treatment Influences the Benefits of the PP Modules in Positive Affect

**FIGURE 2:**
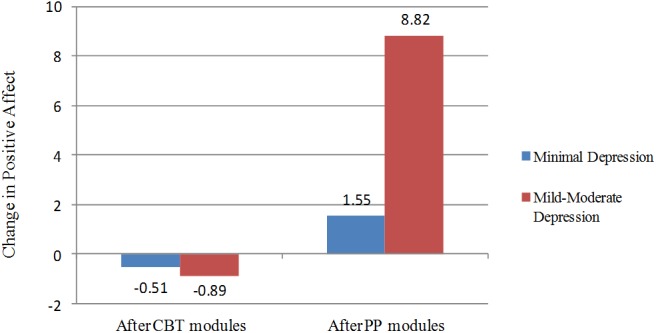
Change in Positive Affect after the CBT modules (M1–M4) and after the PP modules (M5–M8), divided by the level of depressive symptoms. Number of participants with minimal depressive symptoms = 74; Number of participants with mild-moderate depressive symptoms = 29.

Depression severity was rated in terms of BDI-II cut-offs. In this regard, depressive symptoms were clustered into: (a) minimal depressive symptoms (patients with scores from 0 to 13; *N* = 74) and (b) mild-moderate depressive symptoms (patients with scores between 13 and 28; *N* = 29). Outcomes were considered the change in positive affect after the CBT component (M4 minus pre) and after the PP modules (Post minus M4). A MANOVA analysis was conducted with depression severity as independent variable and positive affect outcomes after the CBT modules and after the PP modules as dependent variables. Using Pillai’s trace, there was a significant difference in the change in positive affect between patients with minimal depressive symptoms and those with mild-moderate depressive symptoms [*F*_(2,100)_ = 9.86, *p* < 0.001]. In this regard, separate univariate ANOVAs showed a non-significant change in positive affect between depression severity levels after the CBT modules [*F*_(1,101)_ = 0.06, *p* = 0.80, η^2^*p* = 0.001]. However, the change in positive affect after the PP modules was significantly greater for patients with mild-moderate depressive symptoms compared to patients with minimal symptoms [*F*_(2,100)_ = 15.69, *p* < 0.001, η^2^*p* = 0.13], with a large effect size (Cohen, 1992). These results are depicted in Figure 2.

## Discussion

The main objective of this study was to investigate the evolution of positive and negative functioning outcomes across the eight modules of an Internet-based intervention for depression, and explore the specific contribution of the PP component to the outcomes. Results showed that participants significantly improved their anxiety, depression, and negative and positive affect scores throughout the intervention.

The exploration of the changes over time makes it possible to not only examine the pre-post change, but also to analyze the changes occurring during the intervention depending on the strategies learned throughout the intervention process. In this vein, the change in outcomes was examined before and after the introduction of one of the essential psychological components of the intervention program: the PP component. Results showed that negative functioning measures (ODSIS, OASIS, and PANAS-) decreased significantly during the implementation of the CBT modules, and also during the implementation of the PP modules, with the exception of depression (ODSIS), which only decreased significantly after the introduction of the PP modules. These results suggest that PP techniques might have an impact on the decline in clinical symptomatology, which is consistent with prior studies (Vázquez et al., 2006; Chaves et al., 2017). In the case of positive affect, results showed that there was no improvement after the implementation of the CBT components, but the improvement was significant after the introduction of the PP component. These results emphasize the need to include PP exercises and techniques in conventional treatments to directly address improvements in positive emotions and achieve a more profound change in positive functioning measures (Sin and Lyubomirsky, 2009; Bolier et al., 2013). In this regard, positive emotions can lead to positive behaviors and states of happiness that contribute significantly to robust health, mitigate the physiological and cognitive effects of negative emotions, and decrease reactivity to stress (Fredrickson and Branigan, 2005; Fredrickson, 2006; Bower et al., 2009). Thus, PP relies on the hypothesis that depression can be treated effectively, not only by reducing negative symptoms, but also by building positive emotions, character strengths, and meaning (Seligman et al., 2006). Therefore, it is important to include PP strategies to directly build up these positive resources in order to counteract negative symptoms and buffer against their future reoccurrence, as in the intervention presented here.

Our second goal was to explore whether more depressed patients would show greater improvements in positive affect, compared to those with less severe depressive symptoms. Results showed that participants did not improve positive affect after the CBT component, and no differences were found between the two groups. However, after the introduction of the PP component, patients with mild to moderate depressive symptoms had significantly larger improvements in positive affect, compared to those with minimal depression. Our results are consistent with the findings of a meta-analysis showing that depression severity moderates the effectiveness of PPIs (Sin and Lyubomirsky, 2009). These results could be explained by the fact that patients with mild to moderate depressive symptoms depart from lower levels of positive affect (floor effect) and have more room for improvement. In any case, as Sin and Lyubomirsky (2009) claimed in their meta-analysis, these findings challenge the notion that people with more severe depressive symptoms might benefit less from PPIs, because their affective, behavioral and cognitive characteristics keep them from taking full advantage of the relevant positive activities.

In this regard, the results obtained in the present study suggest that positive clinical psychology, that is, the combination of clinical and positive psychology approaches, can be an effective way to treat depression by reducing negative symptoms and increasing positive emotions (Wood and Tarrier, 2010). The results suggest that this protocol achieved the two goals considered essential in the treatment of patients with depressive symptoms, that is, reducing discomfort and negative emotions and promoting strengths and the increase in positive emotions (Vázquez and Hervás, 2008). An important strength of the present study is that it achieved these goals through an online intervention. The development of Internet-based interventions with a focus on both negative and positive aspects of human functioning will allow a more comprehensive psychotherapy (Rashid, 2009).

This study has limitations. First, the intervention was not compared to another intervention without the PP component, which keeps us from drawing firmer conclusions about the specific contribution of PP. Furthermore, bear in mind that these findings should be interpreted cautiously because a carry-over effect of the CBT component could explain the larger improvements in positive affect after the introduction of the PP component, although these effects are not observed in the other clinical measures. In future studies, we will take into account the importance of counter-balancing the order of the intervention and carrying out studies with dismantling designs in order to discover the specific contribution of the PP component. In this regard, we are currently conducting a research trial designed to dismantle the Internet-based positive clinical psychology program used in the present study, in order to discover the contribution of each specific component (ClinicalTrials.gov ID: NCT03159715). Moreover, it might be relevant to include other PP measures in the present study to investigate how the PP modules affect them. Future studies should also determine whether it is better for the PP component to be placed at the beginning or the end of an intervention. Furthermore, the low symptom severity should be included as one of the limitations because most of the sample were in the mild depressive range. It is important for further studies to explore the effect of PP strategies on severely depressed patients. Lastly, all the participants had to be facing a stressful event at baseline assessment in order to be included in the trial, but the presence of this event was not explored at post-assessment. Therefore, we cannot ensure that this stressor was absent after the intervention, which could potentially affect the results, although new stressful events could also arise.

## Conclusion

In sum, this is the first study to explore the incorporation of a positive psychology component in an Internet-based program across each module. The negative clinical symptomatology decreased significantly during the implementation of both components, namely CBT and PP, with the exception of depression, which only improved during the implementation of the PP component. Likewise, positive affect increased only after the introduction of the PP modules. These results suggest that PP techniques might have an impact on clinical symptomatology, and they emphasize the need to include them to achieve a more profound change in positive functioning measures. Furthermore, the more depressed patients obtained greater improvements in positive affect during the PP strategies, compared to those with lower depressive symptoms. Overall, the present study provides further clinical and scientific support for the integration of positive and clinical psychology (Fava and Ruini, 2003; Rashid, 2009; Wood and Tarrier, 2010) using an Internet-based program. In addition, as more studies in this field emerge, these two approaches will become more unified. If these results are replicated, we speculate that future therapy for depression may combine talking about troubles with understanding and building positive emotions, engagement, and meaning.

## Ethics Statement

This study was carried out in accordance with the recommendations of Ethics Committee of Jaume I University with written informed consent from all subjects. All subjects gave written informed consent in accordance with the Declaration of Helsinki. The protocol was approved by the Ethics Committee of Jaume I University.

## Author Contributions

AM drafted the manuscript with important contributions from AE, JB-L, and CB. AM in collaboration with, JB-L and CB designed the study and participated in each of its phases. ÁE, DC, A-GP, and RB collaborated in the manuscript development and participated in each study phase. All authors participated in the review and revision of the manuscript and have approved the final manuscript to be published.

## Conflict of Interest Statement

The authors declare that the research was conducted in the absence of any commercial or financial relationships that could be construed as a potential conflict of interest. The reviewer YLDH declared a shared affiliation, with no collaboration, with one of the authors, AM to the handling Editor at the time of the review.
